# Comparative study on muscle-tendon stiffness and balance impairment in postmenopausal women: a focus on osteosarcopenia and osteoporosis

**DOI:** 10.1007/s40520-024-02888-3

**Published:** 2024-12-03

**Authors:** Elham Bagheri Yekta, Giti Torkaman, Leila Aghaghazvini

**Affiliations:** 1 Physical Therapy Department, Faculty of Medical Sciences, Tarbiat Modares University, Ale-Ahmad Ave, P. O. Box: 1411713116, Tehran, Iran; 2 Musculoskeletal Imaging Research Center, Shariati Hospital, Tehran University of Medical Sciences, Tehran, Iran

**Keywords:** Osteosarcopenia, Osteoporosis, Shear wave elastography, Postural control, Achilles tendon, Women

## Abstract

**Background and aims:**

This study set out to examine the stiffness of the gastrocnemius medialis (GM) and Achilles tendon across postmenopausal women with osteosarcopenia (OS), osteoporosis (OP), and normal bone mineral density. Furthermore, we explored the relationship between muscle-tendon stiffness and postural sway during a curve-tracking task in both sagittal (AP) and frontal (ML) planes.

**Methods:**

Seventy-three women volunteered to participate in this study. The participants were classified into OS (T-score ≤ − 2.5 and muscle mass below 5.5 kg/m^2^), OP (T-score ≤ − 2.5), and healthy (T-score >-1) groups. The shear wave elastography was used to determine GM and Achilles tendon stiffness during rest and activation. The postural sway was recorded using a force plate during the performance-based curve tracking (CT) task.

**Results:**

The stiffness of the GM and Achilles tendon was found to be significantly lower in the OS group compared to the OP and healthy groups (*P* < 0.05). In the CT task, the OS group exhibited a significant decrease in the mean absolute (*P* = 0.011) and RMS error (*P* = 0.022) in the ML direction compared to the OP group. Additionally, a positive correlation was found between the ML mean absolute error and both GM and Achilles’s stiffness during rest and activation (*P* < 0.05).

**Discussion and conclusion:**

The OS group exhibited the lowest muscle-tendon stiffness. The GM and Achilles stiffness was positively correlated with poor performance-based balance, particularly in the ML direction. This may increase the risk of falls and subsequent hip fractures during simple daily weight- shifting activities in women with osteosarcopenia.

## Introduction

Osteoporosis is a major health problem worldwide, responsible for a serious clinical and financial burden due to the increasing life expectancy. According to the world health organization, osteoporosis is a systemic skeletal disease, characterized by low mass and micro architectural deterioration of bone tissue, leading to enhanced bone fragility and a consequent increase in fracture risk [1]. With age, not only does bone density decrease, but muscle strength and volume also diminish. The reduction in activity and changes in daily activity patterns among the elderly may be linked to the close relationship between bone and muscle in growth and maintenance. This relationship is well described by the concept of the bone-muscle functional unit and muscle-bone interaction [2]. These adverse health events may be related in part to the progressive wasting of muscle-bone tissue giving rise to sarcopenia (progressive loss of muscle mass, strength and physical functioning) [3] and osteoporosis [4]. Appendicular skeletal muscle mass index (ASMI), which has been used for definition of sarcopenia, has been suggested to be positively related to bone mineral density (BMD) [5]. When one of both tissues undergoes the aging process, the function of the other appears to be affected. They also share similar etiological factors such as mechanical and functional influences, genetic aspects, nutritional disorders, chronic inflammation, lifestyle habits, and hormonal changes [6–8].

When an older person presents with both osteoporosis and sarcopenia, a musculoskeletal syndrome termed osteosarcopenia can be identified. The clinical importance of osteosarcopenia is evidenced by the strong association with falls and fractures [9–11]. Reduction of muscle strength and lean mass loss becomes more detrimental in postmenopausal women and can be influenced by several environmental factors, such as low physical activity and inadequate nutrition [5].

Some physiological and functional disturbances, such as decreased muscle mass and strength, slow walking speed, and balance problems, have been reported with both osteoporosis and sarcopenia. However, these issues may be exacerbated in individuals with osteosarcopenia, thereby increasing their risk of falling. Consequently, identifying those suffering from osteosarcopenia has significant clinical implications and can effectively reduce fall risk and prevent bone fractures [12].

In addition, aging is associated with changes in the sonoelastographic properties of muscles and tendons. It has shown that older individuals generally exhibit a decrease in muscle mass and thickness [13]. Additionally, an increase in muscle stiffness has been observed in the rectus femoris and biceps brachii muscles of older adults [14]. However, the literature presents inconsistent findings regarding these changes. For instance, Kwan et al. propose that tendons stiffen with age due to water loss and a more disorganized structure of collagen and elastin [15]. Conversely, Lui and Wong report a decrease in the number and functionality of tendon stem/progenitor cells and disorganized collagen bundles [16]. These conflicting reports contribute to the ongoing debate about the impact of age-related changes in muscle-tendon mechanical properties.

Postural control is a complex process that is related not only to strength and coordination but also to the mechanical properties of muscle-tendons, such as stiffness [14]. Elderly women with osteoporosis often experience increased postural imbalance, which consequently heightens their risk of falling [17, 18]. Rezaei et al. showed that in the curve tracking (CT) task, all quantified parameters, including RMS and area of error, were significantly lower in osteoporotic women compared to non-osteoporotic women [19]. However, in static situations, women with osteoporosis have been described as having greater velocity and more anterior-posterior (AP) and medial-lateral (ML) displacement of the center of pressure sway (CoP) than age-matched women without osteoporosis [20]. It has been suggested that ML postural control is an important factor for fall risk in older adults and that postural control is less stable in the ML direction than in the AP direction [19].

For a more comprehensive understanding of postural control strategies, it is advantageous to consider functional tasks. These tasks encompass complex CoP movement patterns in both AP and ML directions and underscore the role of muscle-tendon units. Therefore, challenging tasks such as CT appear more sensitive in distinguishing balance control strategies between women with osteoporosis and osteosarcopenia. Moreover, functional tests emphasize the importance of muscle strength, coordination, reaction time, and the mechanical properties of muscle-tendon units. These factors are vital for implementing effective control strategies and preventing falls.

Given that individuals with osteoporosis and osteosarcopenia represent high-risk aging populations susceptible to falls, this study aimed to explore changes in the quality and quantity of the gastrocnemius muscle, which plays a critical role in weight-shifting control strategies. Our primary objective was to distinguish these changes among three groups: those with osteoporosis, those with osteosarcopenia, and healthy age-matched individuals. Specifically, we compared the stiffness of the gastrocnemius medialis and Achilles tendon among elderly healthy women and those diagnosed with osteoporosis and osteosarcopenia. Additionally, we evaluated the correlation between muscle-tendon stiffness and CoP sway during the CT in both AP and ML directions.

The potential clinical implications of this study will be significant. By identifying specific changes in muscle and tendon stiffness associated with osteoporosis and osteosarcopenia, our findings will inform clinical practices aimed at reducing fall risk and preventing fractures in these high-risk populations. This will lead to improved diagnostic and therapeutic strategies, ultimately enhancing patient outcomes and quality of life.

## Methods and materials

### Study design

This cross-sectional study was conducted in the motion analysis laboratory of the Physical Therapy Department at Tarbiat Modares University and Ultrasonography Center of Shariati Hospital, Tehran, Iran. Our protocol was approved by the ethics committee of the Tarbiat Modares University (IR.MODARES.REC.1402.038) in accordance with the Declaration of Helsinki’s later amendments. After calculation by G-power software, based on the Onambele^’^s study the sample size was defined to be 21 individuals in two osteoporotic and osteosarcopenic group [21]. 10 person were considered for the non-osteoporotic (healthy) age-matched group.

### Participants

**Fig. 1:**
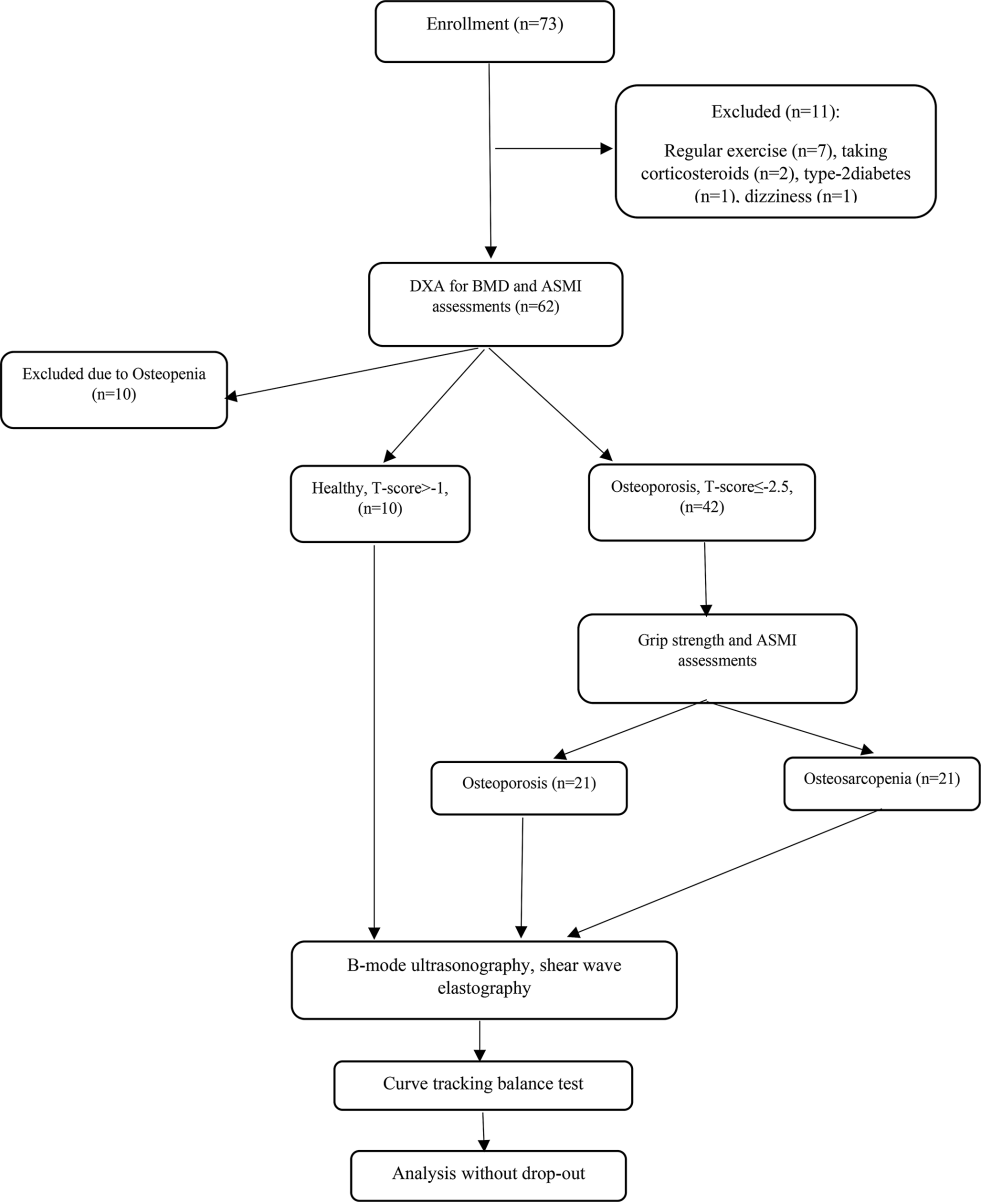
The study flowchart

The study was conducted between March and October 2023 and volunteers were recruited through public notices in rheumatology and bone densitometry departments. The inclusion criteria for participation in the study were women aged 60 years or older without doing regular exercises (2–3 sessions/day for 30 min), and the physical activity scoring according to Baecke questionnaire between 5 and 8 [22]. The exclusion criteria were defined as secondary osteoporosis, osteopenia, musculoskeletal, neurological, and vestibular diseases and other conditions caused instability, diabetes, Alzheimer’s disease, severe cognitive problems (MMSE < 25), cardiovascular/pulmonary diseases, and taking corticosteroids. Based on the predicted sample size, the sampling continued until 21 osteoporotic (OP) and 21 osteosarcopenic (OS) women were recruited. In total, seventy-three volunteers were screened, with eleven excluded for the following reasons: seven engaged in regular exercise, two used corticosteroids, one experienced dizziness, and one had type-2 diabetes (Fig. 1). Those meeting the remaining inclusion criteria (*n* = 62) underwent Dual-energy X-ray absorptiometry (DXA) scans. Subsequently, ten individuals were excluded due to osteopenia (-2.5 < T-score≤-1). Based on the T-score and appendicular skeletal muscle mass index (ASMI), the remaining eligible participants (*n* = 52) were classified into OS (T-score ≤ − 2.5 and ASMI below 5.5 kg/m^2^), OP (T-score ≤ − 2.5 and ASMI more than 5.5 kg/m^2^), and healthy (T-score >-1 and ASMI more than 5.5 kg/m^2^) groups. All participants were informed about the study objectives and methods and provided signed informed consent.

### Procedures

In this cross-sectional study, all assessments were conducted from 9:00 AM to 1:00 PM. The recorded anthropometric variables included age (years), body mass (kg), and height (cm). The body mass index (BMI, kg/m²) was then calculated. The maximum isometric plantar flexion (MIPF) of the ankle was measured three times on the dominant side using a digital handheld dynamometer (Hand-held Dynamometer; Lafayette Instrument Co., Lafayette, IN, USA). Each isometric test lasted five seconds, with one minute of rest between tests. The mean value of the three repetitions was reported as the MIPF. Additionally, the fear of falling was assessed using the fall efficacy scale, FES-I (FES). The Persian version of the FES, which has demonstrated acceptable validity and reliability for the Iranian older adult population, was used [23].

#### Bone densitometry and muscle mass evaluation

Upon obtaining anthropometric data and ensuring adherence to the inclusion and exclusion criteria, volunteer participants underwent DXA scans at the bone densitometry center of Shariati hospital. The HOLOGIC DXA (ASY-00409, USA) device was used to measure bone density in the lumbar vertebrae and neck of the femur, resulting in a T-score determination. According to DXA results, firstly, osteoporotic and healthy women were identified based on their T-scores (T-score≤-2.5 and T-score>-1, respectively). Then, ASMI were considered to identify sarcopenia. ASMI was derived from the combined muscle mass of the arms and legs, serving as a reliable proxy for whole-body skeletal muscle mass [24]. To account for the potential influence of body size or height, skeletal muscle mass variables were calculated as below:

ASMI (Kg/m^2^) = ASM/hight^2^ [25].

The next step is to determine osteosarcopenic cases by performing the algorithm to identify sarcopenia cases based on the European Working Group on Sarcopenia in Older People (EWGSOP2) [3].

Hand grip strength test was measured by Vigorimeter (Martin Vigorimeter, Germany) measuring instrument. The Martin Vigorimeter is a pseudo dynamic dynamometer that measures the pressure when subjects press a rubber bulb connected by a tube to a manometer. The validity and reliability of this method has been proven [26].

After performing DXA and handgrip strength assessments, osteoporotic women were categorized as having sarcopenia if their muscle mass fell below 5.5 kg/m², and their hand grip strength was below 20 kg. Those meeting these criteria were placed in the OS group; otherwise, they were enrolled in the OP group.

#### B-mode ultrasonography measurements

Ultrasound images of the GM muscle were recorded using a B-mode ultrasound imaging device (Mindray, DC80, China), with a wide-band linear probe (L145WE) with a 35 mm wide field of view, and coupling gel (ultrasound gel, Poly gel, Iran) between the probe and skin. Ultrasound settings (Frequency: 12 MHz, Gain: 53 dB, Dynamic Range: 135db) were kept consistent across participants. To ensure consistent imaging of the GM muscle between trials, participants were asked to lie prone with their feet hanging from the edge of the examination table and maintain the ankle joint in an anatomically neutral position (0° of dorsi flexion). They stayed in this position for 10 min to allow fluid shift before the measurements. Ultrasonography was conducted at the fixed point of GM muscle in the middle of two reference points. One of the reference points was at the proximal one-third of a longitudinal line from midway between the medial and lateral malleoli to midway between the medial and lateral epicondyles of the femur. The other point was at the medial end on a transverse line perpendicular to the point on the longitudinal line [27]. The muscle architecture was measured using electronic calipers in real-time B-mode ultrasonography. Muscle thickness (MT), measured at the center of the image from the superficial to deep aponeurosis, in the longitudinal view. Pennation angle (PA), was defined as the angle between the muscle fascicle and the deeper aponeurosis in the longitudinal view [27]. Cross sectional area (CSA) of the GM muscle obtained by extended field of view (EFOV) in transverse view. The CSA of the muscle has been carefully drawn with the freehand drawing tool [28]. Three images (with the probe removed and replaced) were recorded at rest (GM-rest) and during muscle activation (GM-activation, isometric voluntary plantar flexion exertion) on the dominant leg’s GM muscle. The average of the three measurements was used in the subsequent analysis. To assess muscle quality, three images were recorded with the ultrasound device set to default gain levels. Echo intensity (EI), was determined quantitatively using computer-assisted grey-scale analysis. Briefly, the grey-scale analysis was applied after selecting a region- of-interest that comprised the entire muscle without the surrounding fascia. The mean EI of this region was next calculated with a standard histogram function and expressed as a value between 0 (black) and 255 (white) as the ultrasound was created with 8-bit grey-scale [29]. The EI was measured in the three images taken of GM muscle at rest and activation and the mean was taken to reduce measurement variation.

#### Shear wave elastography

**Fig. 2:**
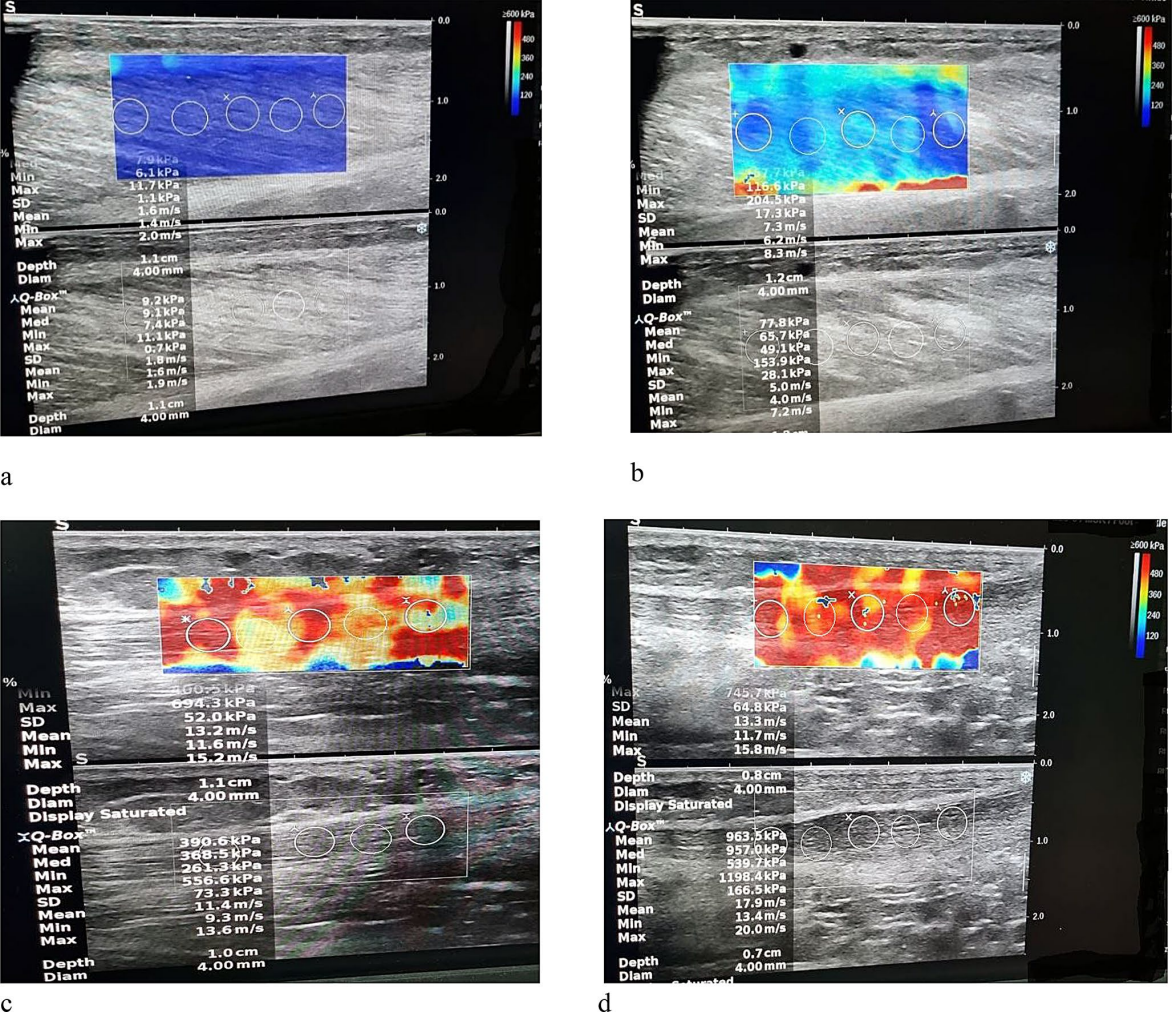
A sample of shear wave elastography of the gastrocenemius medialis (GM) and Achilles tendon. GM during rest (**a**), GM during activation (**b**), Achilles tendon during rest (**c**), Achilles tendon during activation (**d**)

The SWE was used to determine GM and Achilles tendon shear modulus during rest and activation of the plantar flexors (Fig. 2a-d). The SWE relies on the measurement of shear wave velocity that results from mechanical perturbations to the tissue. The SWE has been validated against traditional material testing to provide an estimate of muscle stiffness [30]. Supersonic MACH30 device (Hologic Supersonic Imagine, France) with L18-5 probe were used to take the SWE of the GM and Achilles tendon after finishing ultrasonography. Since regional differences are present along the length of the Achilles tendon [27], all measurements were taken in the middle part of the free tendon, while they were in prone position and their foot and ankle were relaxed for the rest position. After that the subject did ankle plantar flexion and the rest of the pictures were captured. A generous amount of coupling gel was used to enable imaging of the Achilles tendon without applying any pressure. More specifically, during imaging, the ultrasound probe was placed along the longitudinal axis of the tendon. The transducer held still for ≈ 10 s to allow for the elastogram to stabilize before saving a single elastography image [31]. For determination of the SWE for GM the probe was placed on the bulk of the GM like conventional ultrasonography as mentioned before. The ROI for SWE was set within a predefined circular sampling box. The diameter and inspection depth of the ROI were respectively set to 4 mm and 0.5–1.5 cm. Tissue stiffness and velocity were then quantitatively calculated within those ROIs and depicted in kPa and m/s, respectively. All musculoskeletal sonography and SWE assessments were conducted by a single sonographer with five years of experience in musculoskeletal imaging.

#### Performance-based posturography

A computerized force plate recorded CoP fluctuations and relative parameters (9286B, Kistler Co., Winterthur, Switzerland). The Kistler-Mars Software was used to properly analyze postural balance characteristics at dynamic tasks, supporting the Kistler data acquisition system. Using the Kistler-Mars software, the force plate CoP signals acquired in curve tracking task. The participants were asked to stand barefoot in the center of the force plate in a two-legged position while keeping their arms next to the body. An LCD screen showed visual feedback of the CoP displacements and displayed the CoP real-time position and movement. In the CT task, the subject was asked to track the path in a sinusoidal shape. The CoP location was displayed on the LCD screen to guide the task execution. The participant had to follow the sinusoidal reference curve by shifting her weight on her stable fixed feet in a double stance position within 30 s in AP and ML sinusoidal references curve. The mean absolute error values, standard deviation (SD) of absolute error, and root mean square (RMS) error between the reference curve and actual CoP signal were recorded in millimeters. The area between the reference curve and the actual CoP signal was calculated as the integral absolute deviation between the reference and patient tracking curves (mm × s). Each AP/ML sinusoidal references curve was repeated two times with one-minute rest in between, and five minutes rest were given between AP and ML curves. The mean value of two repetition was used for data analysis.

### Statistical analysis

The data’s normal distribution was confirmed using the Shapiro-Wilk test, where the P-value was greater than 0.05. To compare parameters across groups, a one-way ANOVA was employed, followed by a post-hoc Bonferroni test. Given the normal distribution of the data, a two-tailed Pearson correlation coefficient was utilized to examine the relationship between the time-varying balance parameters of the COP, GM, and Achilles tendon SWE. The threshold for statistical significance was set at *P* ≤ 0.050. All statistical analyses were performed using SPSS version 19 (IBM, Armonk, NY, USA).

## Results

Table 1 shows the demographic characteristics, the hip and lumbar T-score, ASMI, and FES in all groups. Height, and BMI showed no significant differences between the groups (*P* > 0.05). The age was significantly lower in the OP and healthy group compared to the OS group (*P* < 0.001 and *P* < 0.001, respectively). The FES score also was significantly lower in the healthy group compared to OP (*p* = 0.025) and OS (*p* < 0.001) groups. It was lower in the OP group compared to the OS group (*p* < 0.001). ASMI were significantly lower in the OS group compared to the OP (*p* < 0.001) and healthy (*p* < 0.001) groups. The comparison of T-scores in the hip region shows that there was no significant difference between the OS and OP groups (*P* = 0.068). There was a significant difference in the lumbar T-score between all groups (between the OS and OP, *P* = 0.004, OS and healthy, *P* < 0.001, and OP and healthy, *P* < 0.001). The MIPF showed a significant decrease in the OS group compared to OP and healthy groups (*P* < 0.001), but it was not significant between the OP and healthy group (*P* = 0.066).

**Table 1:** Demographic characteristics, T-scores, ASMI, and FES in the groups (Mean ± SD)

Variables	OS	OP	Healthy	*P*-value
Age (year)	73.9 ± 7.1^*,#^	64.3 ± 4.4 ^$^	65.3 ± 3.8	<0.001
Height (cm)	155.07 ± 7.0	156.04 ± 4.96	158.05 ± 3.91	0.396
BMI (kg/m^2^)	27.63 ± 4.59	31.45 ± 4.65	31.03 ± 7.65	0.058
T-score Lumbar	-2.9 ± 0.37 ^*, #^	-2.6 ± 0.21 ^$^	1.17 ± 0.21	<0.001
T-score femoral neck	-2.9 ± 0.37 ^*^	-2.68 ± 0.24 ^$^	1.07 ± 0.28	<0.001
ASMI	4.82 ± 0.42 ^*, #^	6.06 ± 0.40 ^$^	7.38 ± 0.98	<0.001
MIPF(Kg)	5.66 ± 1.25 ^*,#^	7.34 ± 1.90	8.73 ± 1.01	<0.001
FES	57.57 ± 5.13^*, #^	18.48 ± 9.30 ^$^	11.30 ± 0.82	<0.001

ASMI: Appendicular skeletal muscle mass, FES: Fall efficacy scale, MIPF: Maximum isometric plantar flexion strength. OP: Osteoporotic group, OS: Osteosarcopenic group. * Significant difference between OS and OP groups, ^#^ Significant difference between OP and healthy groups, ^$^ significant difference between OS and healthy groups

### Ultrasonography and shear wave elastography parameters

The musculoskeletal sonography parameters for the GM in rest and activation modes are illustrated in Table 2. During rest, CSA showed a significant difference between groups (*P* = 0.015). In the OS group, the CSA showed a significant decrease compared to the healthy group (*P* = 0.039). No significant decrease was seen in the OP group compared to the healthy group (*P* = 1.000). Additionally, for MT, there was a significant decrease in the OS group compared to the OP (*P* = 0.004) and healthy (*P* = 0.004) groups; there was no significant difference between the OP and healthy groups (*P* = 1.000). The highest and lowest EI were seen in the OS and healthy groups, respectively; all groups were significantly different from each other (*P* < 0.001). During activation, CSA showed a significant decrease in the OS group compared to the OP (*P* = 0.009) and healthy (*P* < 0.001) groups, but there was no significant difference between the OP and healthy groups (*P* = 0.199). The MT also showed a significant decrease in the OS and OP groups compared to the healthy group (*P* = 0.002 and *P* = 0.003, respectively). The highest and lowest EI were seen in the OS and healthy groups, respectively; all three groups were significantly different from each other (*P* < 0.001).

**Table 2:** The ultrasonography parameters of GM in the rest and activation status (Mean ± SD)

Variables	OS		OP	Healthy	*P*-value	ES
**Rest status**						
CSA(mm^2^)	12.64 ± 0.94^#^	14.04 ± 1.78		14.47 ± 1.76	0.015	0.157
MT(mm)	12.76 ± 0.55^*#^	15.44 ± 2.73		15.76 ± 2.17	0.002	0.232
EI	141.14 ± 21.33^*#^	114.56 ± 5.34^$^		83.95 ± 8.9	<0.001	0.504
**Activation status**						
CSA(mm^2^)	12.28 ± 2.06^*#^	13.85 ± 1.29		13.85 ± 1.29	<0.001	0.303
MT(mm)	12.81 ± 2.39^*,#^	15.62 ± 2. 67		16.07 ± 2.05	<0.001	0.272
EI	123.2 ± 19.04^*#^	98.14 ± 21.1^$^		68.04 ± 8.42	<0.001	0.559

CSA: cross sectional area, EI: echo intensity, ES: effect size, MT: muscle thickness, OP: osteoporotic group, OS: Osteosarcopenic group.*: significant difference between OS and OP, #: significant difference between OS and healthy, $: significant difference between OP and Healthy

**Fig. 3:**
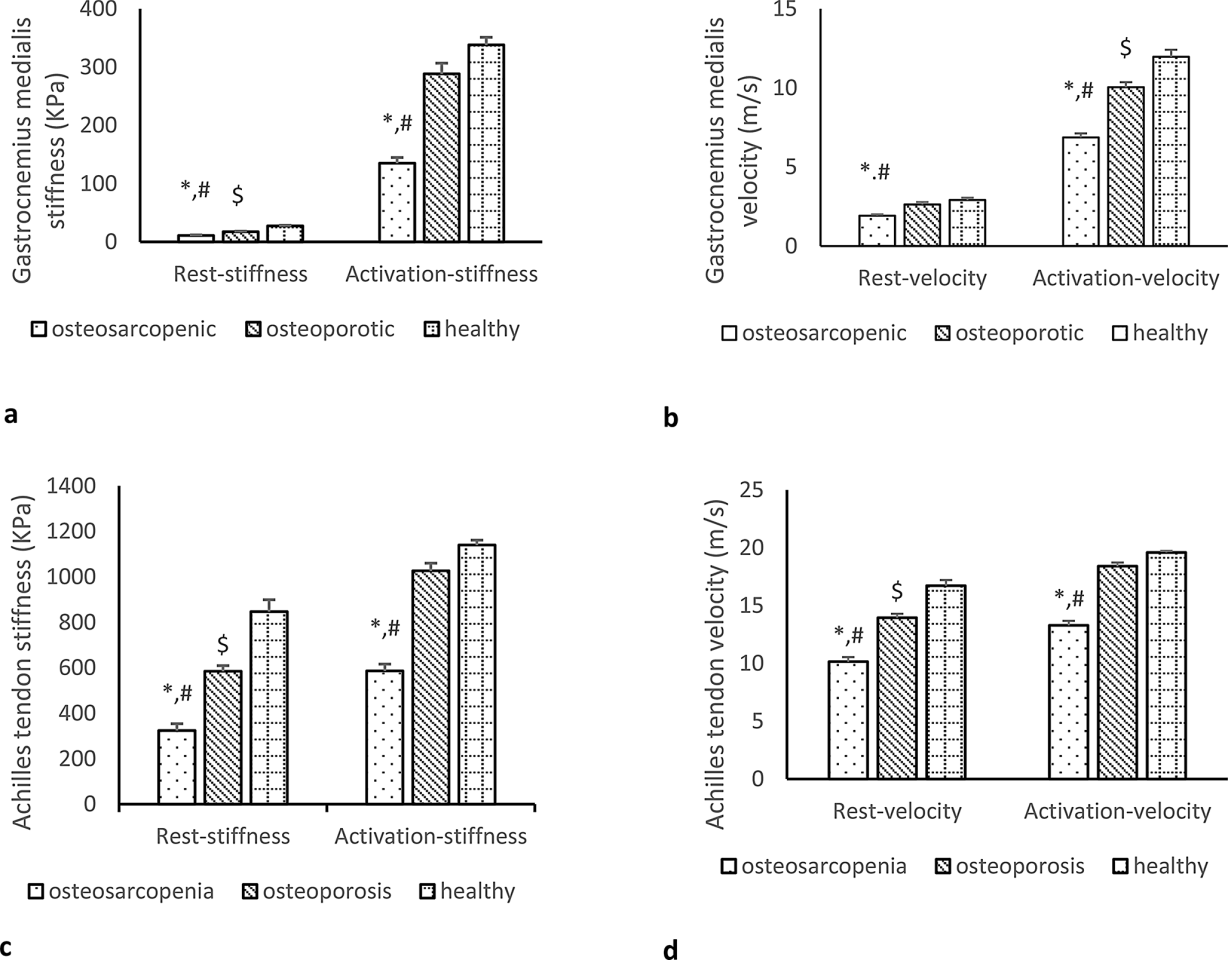
The gastrocenemius medialis (GM) stiffness (**a**), velocity (**b**) and the Achilles tendon stiffness (**c**) and velocity (**d**) in the OS, OP, and healthy groups during rest and activation. * Significant difference between OS and OP groups, ^#^ Significant difference between OP and healthy groups, ^$^ significant difference between OS and healthy groups

During rest and activation, the stiffness and velocity of GM in the OS group were significantly lower than those in the OP (*P* < 0.001) and healthy (*P* < 0.001) groups (Fig. 3a, b). In the OP group, the GM-rest stiffness and GM-activation velocity were significantly lower than those in healthy group (*P* < 0.001); however, GM-rest velocity and GM-activation stiffness showed no significant difference between the OP and healthy groups (*P* = 0.559 and *P* = 0.139, respectively). The stiffness and velocity of the Achilles tendon are illustrated in Fig. 3c, d. At rest, the Achilles tendon stiffness and velocity were significantly lower in the OS group compared to both the OP (*P* < 0.001) and healthy (*P* < 0.001) groups. During activation, there was a significant decrease in the Achilles tendon stiffness and velocity in the OS group compared to the OP (*P* < 0.001) and healthy (*P* < 0.001) groups. In the OP group, the Achilles-rest stiffness and velocity were lower than those in the healthy group (*P* < 0.001). However, there were no significant differences in the Achilles tendon stiffness and velocity during activation between the OP and healthy groups (*P* = 0.099 and *P* = 0.129, respectively).

### Performance-based postorugraphy in AP and ML directions

The CoP displacement during performance-based CT is illustrated in Table 3. In the AP direction only SD of absolute error showed a significant decrease in the OS group compared to that in the healthy group (*P* = 0.021). In the ML direction a significant decrease was seen in the mean absolute error (*P* = 0.033), SD of absolute error (*P* = 0.011), RMS error (*P* = 0.022), and area (*P* = 0.047) for the OS group compared to OP group. These decreases were also significant between the OS and healthy groups (*P* = 0.009, *P* = 0.008, *P* = 0.006, and *P* = 0.012, respectively). No significant difference was seen between the OP and healthy groups.

**Table 3:** The COP sway parameters during the curve tracking task (Mean ± SD)

Variables	OS	OP	Healthy	*P*-value	ES
**AP direction**					
Mean absolute error(mm)	28.67 ± 12.30	35.47 ± 11.39	36.82 ± 8.9	0.86	0.095
SD of absolute error (mm)	18.41 ± 7.44^#^	23.32 ± 7.39	26.16 ± 5.87	0.014	0.160
RMS error(mm)	34.15 ± 14.26	42.54 ± 13.4	45.66 ± 9.04	0.081	0.122
Area(mm*s)	582.96 ± 242.78	709.46 ± 227.9	755.5 ± 153.9	0.080	0.098
**ML direction**					
Mean absolute error(mm)	48.50 ± 17.35^*#^	63.66 ± 17.71	70.85 ± 22.54	0.004	0.198
SD of absolute error(mm)	29.45 ± 10.31^*#^	40.85 ± 12.35	70.85 ± 22.54	0.002	0.219
RMS error(mm)	56.86 ± 20.02^*#^	75.80 ± 21.24	84.25 ± 26.97	0.003	0.212
Area(mm*s)	987.3 ± 343.4^*#^	1273.2 ± 354.3	1418.8 ± 451.7	0.007	0.186

ES: effect size, OP: osteoporotic group, OS: Osteosarcopenic group. *: significant difference between OS and OP, #: significant difference between OS and healthy, $: significant difference between OP and Healthy

### The correlation coefficient between the muscle-tendon shear wave elastography and curve tracking

According to Table 4, in the AP direction of the curve tracking, a significant correlation was observed between the SD of absolute error with the rest GM stiffness (*r* = 0.343), Achilles tendon stiffness(*r* = 0.340), and Achilles tendon velocity (*r* = 0.308). In addition, a significant relationship was seen between the RMS error and the rest-GM stiffness (*r*=-0.301).There were no significant correlation between the mean absolute error and area with the GM and Achilles tendon stiffness and velocity in the rest status (Table 4). A significant correlation was observed between SD of absolute error with the GM velocity (*r* = 0.359), Achilles tendon stiffness(*r* = 0.297), and Achilles tendon velocity (*r* = 0.283) during activation mode. In addition a significant relationship was seen between RMS error and GM velocity (*r* = 0.293).There were no significant correlation between RMS error with the Achilles tendon stiffness and velocity and also the mean absolute error and area with the rest stiffness and velocity of the GM and Achilles tendon in the AP direction.

**Table 4:** The correlation coefficient between shear wave elastography parameters of the GM/ Achilles tendon and curve tracking parameters during rest and activation status in the AP direction, r (P-value)

Variables	GM stiffness	GM velocity	Achilles stiffness	Achilles velocity	
**Rest status**					
Mean absolute error(mm)	0.268(0.054)	0.160(0.256)	0.195(0.162)	0.192(0.173)	
SD absolute error(mm)	0.343^*^(0.013)	0.214(0.128)	0.304^*^(0.029)	0.308^*^(0.027)	
RMS error(mm)	0.301^*^(0.030)	0.185(0.188)	0.240(0.087)	0.239(0.087)	
Area(mm*s)	0.265(0.058)	0.167(0.236)	0.205(0.145)	0.204(0.147)	
**Activation status**					
Mean absolute error(mm)	0.154(0.247)	0.242(0.084)	0.192(0.172)	0.190(0.178)	
SD absolute error(mm)	0.259(0.064)	0.359^*^(0.009)	0.297^*^(0.033)	0.283^*^(0.042)	
RMS error(mm)	0.198(0.158)	0.293^*^(0.035)	0.236(0.093)	0.229(0.102)	
Area(mm*s)	0.162(0.250)	0.254(0.068)	0.197(0.162)	0.190(0.177)	

AP: anterior-posterior, GM: gastrocnemius medialis, RMS: root-mean-square, SD: standard deviation, *: Significant correlation

Based on the Table 5, in the ML direction, a significant correlation was observed between the mean absolute error with the rest-GM stiffness (*r* = 0.311), Achilles tendon stiffness(*r* = 0.393), and its velocity (*r* = 0.385). In addition a significant relationship was seen between the SD of absolute error with the rest-GM stiffness (*r* = 0.317), Achilles tendon stiffness(*r* = 0.421) and its velocity (*r* = 0.422). A significant correlation was also observed between the RMS error with the rest-GM stiffness (*r* = 0.318), Achilles tendon stiffness(*r* = 0.409), and its velocity (*r* = 0.404). Furthermore a significant relationship was seen between the area and the GM rest stiffness(*r* = 0.294), Achilles tendon stiffness(*r* = 0.385) and its velocity(*r* = 0.379).There were no significant correlation between the mean absolute error, SD of absolute error, RMS error and area with the rest-GM velocity, whereas all correlations were significant in the activation status. In the activation mode, there was a significant correlation between mean absolute error of the GM activation stiffness and its velocity, Achilles tendon stiffness and its velocity(*r* = 0.307, *r* = 0.410, *r* = 0.367, and *r* = 0.361 respectively). SD of absolute error also showed a significant correlation with the GM activation stiffness and its velocity, Achilles tendon stiffness and its velocity(*r* = 0.327, *r* = 0.421, *r* = 0.411, and *r* = 0.401, respectively). RMS error showed a significant correlation with the GM activation stiffness and its velocity, Achilles tendon stiffness and its velocity(*r* = 0.322, *r* = 0.424, *r* = 0.387, and *r* = 0.380, respectively). In addition the area also showed a significant correlation with the GM activation stiffness and its velocity, Achilles tendon stiffness and its velocity (*r* = 0.294, *r* = 0.398, *r* = 0.354, and *r* = 0.345, respectively).

**Table 5:** The correlation coefficient between shear wave elastography parameters of the GM/ Achilles tendon and curve tracking parameters during rest and activation status in the ML direction, r (P-value)

Variables	GM stiffness	GM velocity	Achilles stiffness	Achilles velocity	
**Rest status**					
Mean absolute error(mm)	0.311^*^ (0.025)	0.218(0.121)	0.393^*^(0.004)	0.385^*^(0.005)	
SD absolute error(mm)	0.317^*^(0.022)	0.230(0.102)	0.421^*^(0.002)	0.422^*^(0.002)	
RMS error(mm)	0.318^*^(0.021)	0.226(0.107)	0.409^*^(0.003)	0.404^*^(0.003)	
Area(mm*s)	0.294^*^(0.034)	0.212(0.132)	0.385^*^(0.005)	0.379^*^(0.006)	
**Activation status**					
Mean absolute error(mm)	0.307^*^(0.008)	0.410^*^(0.009)	0.367^*^(0.008)	0.361^*^(0.009)	
SD absolute error(mm)	0.327^*^(0.002)	0.421^*^(0.003)	0.411^*^(0.002)	0.401^*^(0.003)	
RMS error(mm)	0.322^*^(0.005)	0.424^*^(0.005)	0.387^*^(0.005)	0.380^*^(0.005)	
Area(mm*s)	0.294^*^(0.010)	0.398^*^(0.012)	0.354^*^(0.010)	0.345^*^(0.012)	

GM: gastrocnemius medialis, ML: medial-lateral, RMS: root-mean-square, SD: standard deviation. *: Significant correlation

## Discussion

This study was designed to compare the quality and quantity of the GM among women with osteosarcopenia, osteoporosis, and healthy age-matched controls. Additionally, we compared the stiffness of the GM and Achilles tendon among these three groups. Furthermore, we evaluated the correlation between GM and Achilles tendon stiffness and CoP sway during the CT in both AP and ML directions.

Based on the research methodology, the main finding was that the structural parameters, including CSA, MT, and EI, showed significant differences between the three groups in both resting and activation modes. A significant observation was that all parameters in the OS group demonstrated a substantial decrease compared to the healthy and OP groups, both at rest and during activation. The only exception was the CSA, which in the rest state showed a significant difference only between the OS and healthy groups. This suggests that musculoskeletal sonography, performed during muscle activation, can offer valuable insights into the muscle’s mechanical output and the level of energy consumption [32]. Furthermore, it may be more effective in comparing and differentiating muscle statuses. However, in the OP group, only the EI showed a significant increase when compared to the healthy group during both rest and activation states. These findings suggest two key points: firstly, they highlight the structural changes in the gastrocnemius muscle in women with osteosarcopenia, even when compared to those with osteoporosis. Secondly, they underscore the importance of EI as a valuable tool in detecting muscle changes. It has been shown that EI independently contributes to muscle strength in elderly individuals [33]. To our knowledge, there are no studies that have investigated the differences between OS and OP groups, but some epidemiological studies have shown the association between sarcopenia and incident osteoporosis [34, 35]. This issue underscores the need for this group to receive more comprehensive screening and treatment. Furthermore, regular screenings are effective in assessing muscle status, a crucial element in balance control. Such screenings can also play a pivotal role in preventing muscle deficiency in postmenopausal women with osteoporosis. Studies have shown that increased proportion of non-contractile elements can be observed during aging by enhanced skeletal muscle EI [36]. Lopez et al. indicates that EI may be an alternative tool for screening of muscle impairment that leads to decreased functional performance in older population [37]. Yoshiko et al. divided all participants (73.7 ± 2.8 years) in to three groups based on EI value of thigh muscles. Their results showed that participants in the higher EI group (lower muscle quality) had a smaller muscle mass, slower gait ability and lower physical activity than participants in the mid EI and low EI groups [38]. Osawa et al. also showed that EI, but not MT, was significantly associated with timed up and go test in very old individuals(88–92 years) [39].

Based on the ultrasonography findings, both at rest and during activation, the GM stiffness and velocity in the OS group were significantly lower than those in the OP and healthy groups. However, the OP group only exhibited lower resting stiffness and activation velocity compared to the healthy group. The Achilles tendon in the OS group displayed the lowest stiffness and velocity during both rest and activation. This observation can be attributed to the fact that individuals with sarcopenia likely have a more severe condition in terms of muscle architecture, with significant differences even in the resting state [40]. Furthermore, the findings underscore the importance of musculoskeletal ultrasonography during muscle activation, in addition to rest. A recent study has also demonstrated that there is no significant difference in the resting state stiffness of the GM muscle between elderly individuals who have experienced falls and those who have not. However, a significant difference was observed during activation [41].

The analysis of CT parameters revealed that the OS group had significantly the lowest area, RMS, and absolute error in the ML direction compared to the OP and healthy groups. In the AP direction, only the SD of absolute error was lower in the OS group compared to healthy subjects. This functional behavior suggests a stiffening strategy to control the CoP sway to prevent falls in conditions where muscle strength, ASMI, and BMD were severely decreased, and the FES was increased in the OS group. Importantly, this change of strategy can be observed in the frontal plane and may indicate a lateral fall in this high-risk group, which increases the risk of femur and hip fractures [42].

Retraining and strengthening muscles, with an emphasis on ankle and hip muscles (due to their crucial roles in the control strategy for fall prevention), preferably in functional exercises with weight bearing, may prevent sudden falls. It should be noted that in the OS group, the use of auxiliary tools such as a harness should be employed to ensure the safety of the exercises. Similar to the present findings, some studies have concluded that the most apparent difference between non-OP and OP concerning CoP control at steady-standing conditions is mainly on the frontal plane [43, 44]. Mofid et al. reported that BMD reduction can affect postural instability in the side-to-side direction [45]; Rezaie et al. also showed that weight shifting and dynamic postural control could alter in the ML direction in postmenopausal women [19].

Given the significance of gastrocnemius muscle function in ankle strategy and activities involving weight shift [44], this study explored the correlation between muscle stiffness, Achilles tendon stiffness, and balance parameters in the CT task. The correlation was observed across numerous parameters. However, two points stood out: firstly, a decrease in muscle and tendon stiffness was primarily associated with a reduction in error quantity in the CT task especially, in the ML direction. Secondly, this positive correlation was predominantly significant with the stiffness of the GM and Achilles tendon during activation mode. According to the results of the previous studies, this behavior may be attributed to the significance of postural sway in the ML direction and the rationale behind increased displacement in this direction being identified as a predictor of falling [44, 46]. The reduction of muscle and tendon stiffness is an age-related phenomenon, with a decrease in muscle activity and contraction playing a significant role in exacerbating it [47]. As bone mineral density and muscle strength decrease, and considering the interplay between these two systems, a shift in control strategies occurs. This shift can be observed as a transition from ankle to hip strategy [48]. The weakening of the ankle strategy leads to further weakening of the leg muscles, particularly the gastrocnemius and soleus muscles, which inevitably results in a decrease in muscle and Achilles tendon stiffness. The muscle weakness and reduction of muscle-tendon stiffness cause instability in performance-based activities including weight shift (such as CT task), which increases the risk of falling and subsequent fractures in daily activities. Rehabilitation programs may play a crucial role in screening, evaluating, and prescribing functional and controlled muscle exercises. These programs can effectively improve control strategies to maintain functional stability and prevent falls, a factor that should be considered in future studies. To compare the effects of sarcopenia and osteoporosis, both individually and combined, on functional balance and CoP sway in postmenopausal women, it is recommended that future studies also investigate groups with sarcopenia, both with and without osteoporosis.

### Limitations

In our study, we utilized the FES questionnaire but did not assess the self-efficacy scale. Given the significance of self-confidence in fall risk, future research should incorporate this measure. Additionally, investigating the association between muscle-tendon stiffness and postural balance in both faller and non-faller subgroups would enhance our understanding. Simultaneous recording of muscle activity during the CK task allows for a more detailed examination of muscle-tendon stiffness and motor unit recruitment, which was not feasible in our current study. Furthermore, it is recommended to explore muscle-tendon stiffness and dynamic balance in osteoporotic and osteosarcopenic groups following a functional-balance exercise program.

While ultrasound is a valuable tool for assessing muscle and tendon properties, it has limitations in terms of reproducibility. Factors such as operator dependency, probe positioning, and tissue anisotropy can affect measurement consistency. To mitigate these issues, we employed standardized protocols and trained operators to ensure consistent probe placement and measurement techniques. Despite these efforts, some variability remains inherent to ultrasound assessments, which should be considered when interpreting our findings. It is recommended to explore muscle-tendon stiffness and dynamic balance in osteoporotic and osteosarcopenic groups following a functional-balance exercise program.

## Conclusions

Based on the results of SWE at rest and during activation, the OS group exhibited the lowest stiffness and the slowest muscle-tendon velocity compared to both the OP and healthy groups. Muscle-tendon stiffness during activation demonstrated a positive correlation with poor balance performance, particularly in the ML direction. These findings emphasize the importance of assessing stiffness during activation mode. Furthermore, they suggest that a decrease in GM and Achilles tendon stiffness could lead to a further reduction in the role of the ankle strategy, resulting in increased instability during dynamic activities such as weight shifting. All these factors heighten the risk of falls and subsequent fractures during daily activities in postmenopausal women with osteosarcopenia.

## Data Availability

No datasets were generated or analysed during the current study.
